# Mind-wandering, or the allocation of attentional resources, is sleep-driven across childhood

**DOI:** 10.1038/s41598-018-37434-5

**Published:** 2019-02-04

**Authors:** Karen Spruyt, Vania Herbillon, Benjamin Putois, Patricia Franco, Jean-Philippe Lachaux

**Affiliations:** 1 Lyon Neuroscience Research Center, INSERM U1028-CNRS UMR 5292 – Waking Team, University Claude Bernard, School of Medicine, Lyon, France; 2 Epilepsy, Sleep and Pediatric Neurophysiology Department, University Hospitals of Lyon, Lyon, France; 3 Lyon Neuroscience Research Center, INSERM U1028-CNRS5292 - Brain Dynamics and Cognition Team, University Claude Bernard, School of Medicine, Lyon, France

## Abstract

Mind-wandering or the spontaneous, uncontrolled changes in the allocation of attention resources (lapses) may cause variability in performance. In childhood, the relationship between the activation state of the brain, such as in attentional performance, and sleep has not been explored in detail. We investigated the role of sleep in attentional performance, and explored the most important parameters of their relationship. We objectively measured momentary lapses of attention of 522 children and correlated them with sleep schedules. In the subgroup of young children (age 7.1 ± 0.6 years; 60.8% girls), increasing age, long sleep duration and assessment closer to the previous night’s sleep period was associated with impaired performance speed and consistency. From pre-adolescence (age 9.4 ± 0.8 years; 50.5% girls) onwards somno-typologies may develop. As a result, in adolescence (age 13.4 ± 1.2 years; 51.3% girls) not only sleep duration but also sleep midpoint and sleep regularity influence the individual speed and stability of attention. Across development, regularity of sleep, individual sleep midpoint and bedtime become increasingly important for optimal performance throughout the day. Attentional performance and sleep shared almost half of their variance, and performance was sleep-driven across childhood. Future studies should focus on intra- and inter-individual differences in sleep-wake behavior to improve performance or decrease mind-wandering in youth by targeting sleep habits.

## Introduction

In the scientific literature, terms such as arousal, alertness, vigilance and attention are often interchangeably used. Despite their varied associations and definitions, these different terms describe the ‘activation states’ of the cerebral cortex. Attention, for instance, is considered to have a significant impact on a variety of neurobehavioral task performances; i.e., it functions as a filter to select stimuli and is therefore an important step in a cascade of interacting neural and neurotransmitter systems. Alternatively, the inhibition of neural networks involved in the regulation of ‘activation states’ of the brain such as the ascending reticular activating system in the brainstem, allows sleep. Sleep and waking, and their characteristic states, therefore depend on activation and inhibition of neural networks^1^.

Pediatric sleep research is a rapidly growing field, with increasing interest shown to the interface of sleep-wake behavior during childhood. For example, polls^2,3^ indicate that nowadays children sleep much less than what is appropriate for their stage of development. Sleep duration of 5–18 year olds has been consistently declining over the last 100 years. Yet this fact remains vigorously debated^4^. In addition, attention problems affect 5.29% of children and adolescents worldwide^5^. Foremost, sleep problems are common in children with attention deficit hyperactivity disorder^6–9^. Both sleep problems and attention problems have been predictive of scholastic underperformance and behavioral maladjustment. Despite a variety of studies investigating the relationship between sleep and attentional behaviors, their association is complex across childhood. A recent meta-analysis reported that sleep loss was not associated with processing speed and attention in childhood^10^; in contradistinction, other studies reported that attention appears to be sensitive to sleep deprivation in adolescents but attentional components of tasks showed inconsistent results to no effects^11^.

Objective evidence of impaired attentional performance due to poor sleep in children is indeed limited (i.e., about 20 studies)^12–18^. Studies often applied an AB design involving ‘an’ experimental change in sleep duration during childhood. Several examples are: one-night sleep loss^12,19^; an average of 30-min sleep extension or restriction successfully accomplished in 60% of 4^th^ and 6^th^ grade children^20^; a 3 week-long sleep schedule manipulation consisting of baseline (self-selected), optimized, and restricted while attending school^14,15^; sleep restriction for 1 week of 1-hour per day in 6 school-aged children^16^; a 5-hour restricted time in bed in 14 school-aged girls^21^; and more recently a randomly counterbalanced within-subjects cross-over design of a 6.5 hour sleep duration in adolescents^22^. Each of these studies suggests that performance is adversely affected by experimentally manipulated sleep schedule conditions, across a variety of neurobehavioral tasks assessing, for example, attention, cognition, school performance and others.

Small effect sizes have been reported regarding the impact of sleep quality, sleep duration and sleepiness on school performance in children and adolescents^23^. Amongst these, sleepiness showed the strongest relation to school performance. Alternatively, a meta-analysis in late adolescence and adulthood yielded small overall effect sizes ascribed to chronotypes, suggesting that evening orientation is associated with poorer academic performance^24^. Although, change towards eveningness is considered to occur mainly at the age of 12–13 years^25^. Arbabi *et al*.^26^ furthermore showed that already at the age of 10 years, evening orientation adversely affected academic achievement.

Other sleep researchers^27^ have proposed that the timing of sleep and wakefulness is more strongly correlated to performance than total sleep time with the addition, that a time of day effect, denoted by a performance increase during the morning, a post-lunch fall, and then an increase in the afternoon, was observed^28,29^. This notion of time of day has been put to practice in terms of school starting times in France, and elsewhere^29–33^. Although authors did not consistently incorporate bedtime, sleep duration or chronotype in their design, they did observe inter-individual patterns in sleep-wake behavior and suggested a flexibility to adjust to different schedules around the age of 10^32,33^.

In other words, pediatric sleep is a complex behavior to study (e.g., inferred from sleep duration, chronotype, bedtime, sleepiness, and other sleep parameters)^34^. Given the diversity in the applied methodologies in the aforementioned studies, and despite all being suggestive of poor(er) performance, neither the overlap with sleep nor the key determinants in performance, are conclusive. To date, neuroscience has focused mostly on daytime functioning (e.g. attention, learning) while the critically important role of sleep in this context has not always been sufficiently considered. At the same time, sleep researchers may have overlooked the shared variance between ‘activation states’ of the brain^35^.

Since the 1960’s it became evident that any cognitive task requires not only the activation of specific brain regions relevant to the task, but also the deactivation of brain regions irrelevant to the task, that might interfere with task-related regions. Lately, accumulating evidence suggests that within the same individual everyday task performance can vary tremendously^36,37^. As a result, the concept of “default-mode” brain activity state or wakeful rest of the brain is gaining empirical support^38–43^. Studies further suggest that some operations of this default system might continue during sleep, might be modulated by sleep, or might be maintained due to sleep^38,39^. At the basis of abnormal resting-state brain activity might be momentary lapses of attention; that is, this wandering of the mind possibly generates variability in performance. Gujar *et al*.^44^ showed that one-night sleep deprivation in adults impaired stability and balance of task-related deactivation in key default-mode regions. That is, subjects showed unsuccessful memory encoding attempts. Such findings concur with conceptions explaining time on task difficulties^45^, difficulties on tasks with higher cognitive load or processing demands^16^, that primarily occur in sleep deprivation experiments. This mind-wandering also coincides with Carskadon’s *et al*.^12^ pertinent observation in children that ‘sleepiness’ is the clearest and most consistent result of sleep restriction. To date, the objective investigation of this “default-mode” brain activity state in relation to sleep and attentional performance across childhood remains unexplored.

The main aim of the current study was to investigate the relationship between sleep and attentional performance in children aged 6 to 18 years old in their natural habitat. We were additionally interested in which of the parameters of sleep and performance where most prominent in this relationship. We hypothesized that impaired attentional performance depends on sleep duration, sleep irregularity with a shift, around pre-adolescence, towards the importance of sleep midpoint. These parameters may further amplify the individual variability in performance.

## Methods

### Procedure

This study was approved by the Institutional Review Board of Rhône-Alpes, Lyon (France), and participating schools. The study was performed in accordance with relevant guidelines and regulations. We obtained informed consent from parents and assent from children. Subjects were 6 to 18 years of age and lived in Lyon. Typically developing children from the 1^st^ to 12^th^ grade were recruited in schools through simple random sampling. That is, none of the children was receiving an Individual Education Plan at school, indicative of significant learning or other difficulties. Children were assessed in small groups of 3 to 5 whenever convenient in their school schedule, i.e., per child the time of assessment was random yet recorded. Assessments were performed during the school year (i.e., no assessments during summer holiday that is July and August).

### Measures

#### Sleep schedule

During the assessment period in the schools, parents filled out health-related questionnaires which provided the bedtime (BT) and wake up time (WU) during the week and weekend (i.e., Saturday and Sunday). We calculated the sleep period time (SPT) for week and weekend. The difference in SPT between weekend and week, approximating sleep irregularity or social jetlag, was also calculated. A higher positive difference means longer SPT in weekend. Chronotype was expressed as midpoint of sleep (SM) which represents the actual timing of daily sleep as a tendency of morningness-eveningness, and is considered as a biological marker in adolescence^46^.

#### Stabilo

Stabilo is an attention test performed on a tablet (several papers in preparation, www.agence-nationale-recherche.fr/Projet-ANR-10-BLAN-1409). It was designed to measure spontaneous, uncontrolled changes in the allocation of attention resources or momentary lapses of attention (MLA). More specifically, subjects perform an attention-demanding task^47^ involving the presentation of a letter at a central fixation point (the target) for 250 ms. The mask (i.e., 500 ms–4 dots) is replaced with a 2 × 2 array of letters that will stay on screen until the subject presses a button. The response can be ‘target letter was present’ button or ‘the target letter was absent’ button. Response buttons were reversed to the subject’s hand laterality (e.g., for the right-handed child, the ‘yes’ button was on the left side of the tablet). The target was present in 50% of the trials. Its presence and position were randomized across trails. Subjects are encouraged to answer as fast as possible due to a challenge-mode design (i.e., improving past performance). Reaction time (RT) is defined as the latency between the onset of the target and the button press. This task involves the following neuropsychological functions^48,49^: a letter encoding, visual search and motor transform.

Four versions have been developed. The standard version includes 50 trials or 2 minutes having a right/wrong auditory feedback with 5 seconds penalty for mistakes. A similar version for children younger than 8 years old, consists of having the first letter of the array always being a V or T. In the colored version, similar to the standard version, the target is always colored in red. The long version includes 200 trials or 10 minutes without auditory feedback or coloring.

Momentary Lapses of Attention (MLA) indexes: Several indexes assessing MLA, or the speed and stability of attention are calculated. A pre-test establishing a subject’s baseline performance; i.e., the fastest response five times in a row without mistake generates a *norm index*. Below this index, a subject is considered guessing. A higher norm index means slower RT. The *speed of attention* is inferred from two indexes: a *best speed index*: a subject’s best RT and a *consistent speed index*: a subjects best RT on 5 consecutive correct sets, i.e., the subject can’t go faster without making mistakes. Higher scores on the speed of attention indexes suggest slower RT and slower RT for a prolonged period, respectively. The *stability of attention* is based on a moving curve of RT (correct) performance. In short, a *precision index* (expressed in percentage) at 3000 ms (*good precision index*) and at 1500 ms (*very good precision index*) can be calculated. For example, to score a 100% subjects need to make no mistakes and respond each time below 3000 ms. Higher scores reflect few mistakes under fast RT (subject’s performance is precise) and faster RT (subject’s performance is very precise), respectively. A *regularity index* is also calculated on a moving curve of (correct) response performance. A *good regularity index* is based on the cut-off of 40%, and a *very good regularity index* uses the cut-off of 20%. That is, subjects scoring higher than for instance 40% succeeded in performing long sets with little RT variability. Higher scores mean long sets with little (subject’s performance is regular) and very little variation in RT (subject’s performance is very regular), respectively. The *error index* represents the percentage of mistakes made during the task.

Parameters calculated based on sleep schedule and daytime assessment: The time of assessment during the day (HRtest) was collected. We calculated the time up to HRtest from WU (i.e., Time since WU), SM (i.e., Time since SM) and BT (i.e., Time since BT). That is, larger values are indicative of a later time of assessment during the day.

### Statistical Analysis

**Figure 1:**
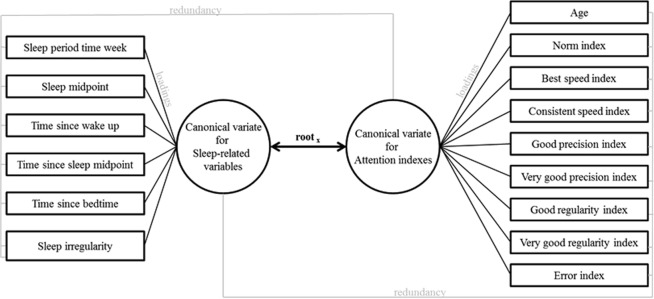
Scheme of variables used. You can think of canonical variates as describing some underlying “latent” variable. The shared variance is the average proportion of variance extracted by the roots. The specific loadings can be found in Table 2. Higher loadings will have more importance within the canonical variates and especially in the root. The redundancy coefficients (i.e., how redundant one set of variables is, given the other set of variables) should be interpreted as a measure of possible predictive ability.

**Figure 2:**
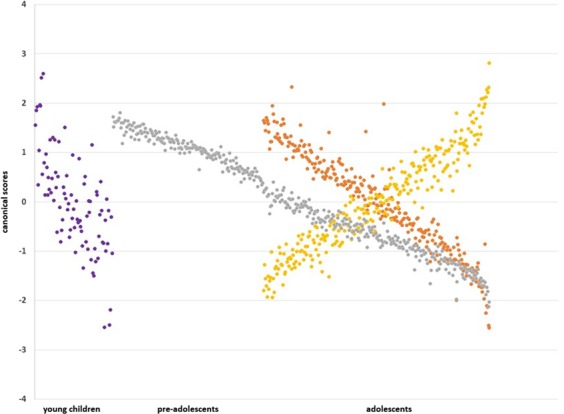
Canonical scores across the age-ranges studied. *Y- axis*: A canonical score is the individual weighted z-transformed score on the set of parameters studied, i.e., sleep and attentional performance; *X- axis*: increasing age; *Purple dots*: 2 minutes standard version for <8 year olds; *Orange dots*: 2 minutes standard version; *Yellow dots*: 10 minutes version of Stabilo; *Grey dots*: 2 minutes standard version for a posteriori regrouping of ≥8 year olds.

**Figure 3:**
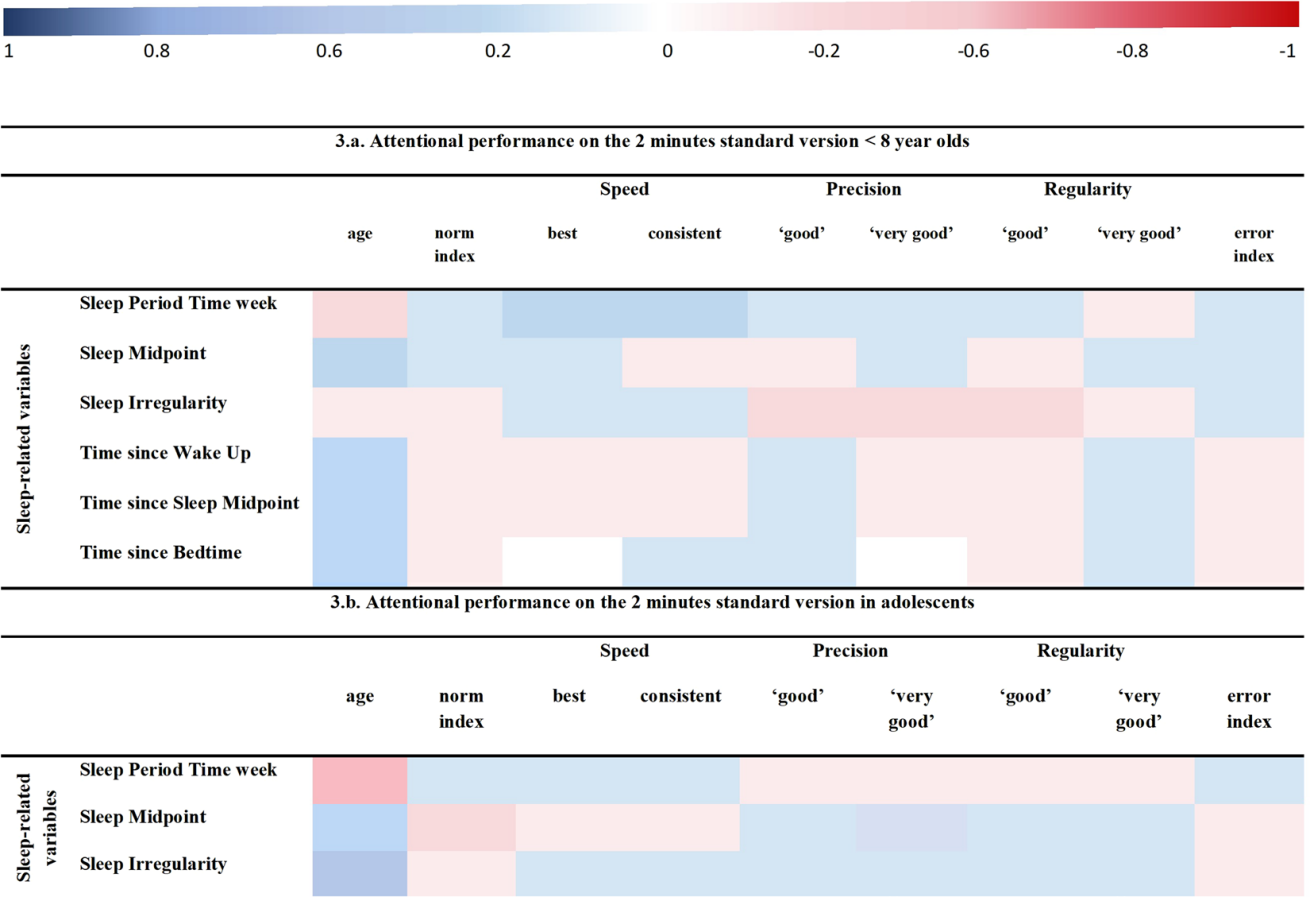
**(3a and 3b)** Correlograms of the CCA performed.

Descriptive analyses were performed to describe the sample. Canonical correlation analyses (CCA) were applied to investigate the concurrent relationship between the group of sleep variables and the group of attention variables. CCA needs to be understood as “multiple-multiple” correlations or extracted roots whereby sets of variables are associated. Figure 1 is a scheme of the variables used for the CCA and a guideline to their interpretation. In addition to the shared variance, the canonical factor loadings (i.e., pertaining to the overall correlation of the variables with the canonical variate or root), lambda prime (i.e., the proportion of unexplained variance) are reported. Loadings could be perceived as expressing the contribution to the shared variance, whereas a canonical score is the individual weighted z-transformed score on the set of parameters studied (Fig. 2). The redundancy index is an index to measure the degree to which one set of variables can predict another set of variables. Inspection of scatterplots (not printed) generated from the CCA’s assists in a more detailed interpretation of the “multiple-multiple” correlations. In addition, correlograms (Fig. 3) may aid the interpretation but caution is warranted since a root is their aggregated ‘result’. Statistical analyses were performed with Statistica version 13 (StatSoft, Inc. (2009), STATISTICA, Tulsa, OK). P-values are determined.

### Statement of Significance

The concept of the brain’s default-mode activity, or wakeful rest, is gaining empirical support. We investigated the degree of commonality between attentional performance and sleep, and explored their determinants by assessing momentary lapses of attention. During childhood, performance was found to be sleep-driven. Especially from pre-adolescence onwards intra- and inter-individual differences in sleep-wake habits may promote mind-wandering, i.e. the spontaneous, uncontrolled changes in the allocation of attention resources. That is, regularity of sleep, individual sleep midpoint and bedtime become increasingly important for optimal performance throughout the day. When non-optimized to the individual need, they may amplify variability in performance. Somnotypology may lead to better understanding of an interdependence between brain activity in the sleep and waking-states.

## Results

Based on the assessment with different versions of Stabilo across the age-ranges included in the study, we report below the results of several subsamples. For reference, we show in Table 1 the sleep parameters of these subsamples. CCA results are included in Table 2, expressing the loadings between the variate and the variables in each set (or correlograms are printing the first-order correlations in Fig. 3a–d). Figure 2 shows the associative trend between the sleep and attention variable sets, expressed as canonical scores, across the age-ranges studied.

**Table 1:** Sleep parameters of the subsamples (mean ± SD).

Sleep parameters (hr)	Young children 7.1 ± 0.6 years old [min. 6 to max. 7.9] (n = 89)	Pre-adolescents 9.4 ± 0.8 years old [min. 8 to max. 10.9] (n = 173)	Adolescents 13.4 ± 1.2 years old [min. 11 to max. 18] (n = 260)
Bedtime week	20.58 ± 0.53	20.77 ± 0.58	21.53 ± 0.77
Wake up week	7.22 ± 0.43	7.08 ± 0.41	6.79 ± 0.42
Sleep Midpoint	2.73 ± 0.58	3.16 ± 0.65	3.80 ± 0.85
Sleep Period Time week	10.64 ± 0.56	10.31 ± 0.66	9.25 ± 0.88
Time since Wake Up	4.97 ± 2.7	4.69 ± 2.49	4.90 ± 2.50
Time since Sleep Midpoint	9.46 ± 2.61	8.61 ± 2.59	7.89 ± 2.52
Time since Bedtime	15.62 ± 2.68	15.00 ± 2.61	14.16 ± 2.46
Sleep Irregularity	0.31 ± 0.64	0.41 ± 1.02	1.39 ± 1.29

Hr: decimal hours format.

**Table 2:** Shared variance of sleep and attention in school-aged children.

	Young children	Adolescents		≥8 year olds standard version
		standard version	long version	
**Sleep-related variables**				
Sleep Period Time week	**0.68**	**0.93**	**−0.93**	**0.95**
Sleep Midpoint	−0.22	**−0.60**	**0.60**	**−0.66**
Sleep Irregularity	0.05	**−0.51**	**0.48**	**−0.66**
Time since Wake Up	**−0.59**	−0.21	0.18	−0.11
Time since Sleep Midpoint	**−0.55**	0.02	−0.05	0.19
Time since Bedtime	**−0.45**	0.12	−0.15	**0.25**
variance extracted	22.9%	25.8%	25.1%	31.4%
redundancy	9.2%	8.1%	7.8%	14.1%
**Attention variables**				
Age	**−0.70**	**−0.96**	**0.96**	**−0.99**
Norm index	0.17	**0.26**	**−0.27**	**0.52**
***Speed***				
Best speed index	**0.38**	0.07	−0.08	**0.28**
Consistent speed index	**0.44**	0.05	−0.07	**0.39**
***Precision***				
Good precision index	−0.09	−0.19	0.17	**−0.49**
Very good precision index	−0.04	**−0.26**	**0.28**	**−0.58**
***Regularity***				
Good regularity index	−0.06	−0.17	0.13	**−0.41**
Very good regularity index	**−0.26**	−0.19	0.11	**−0.39**
Error index	0.13	0.20	−0.08	**0.28**
variance extracted	10.6%	13.6%	12.9%	27.3%
redundancy	4.2%	4.3%	4.0%	12.3%

Canonical Factor Loadings.

In bold canonical factor loadings with weight ≥ ± 0.3.

In young children (age 7.1 ± 0.6 years [min. 6 to max. 7.9]; 60.8% girls) sleep and attentional performance shared 40.3% variance [Chi²(54) = 83.5, p = 0.006, lambda prime: 35.2%]. Their association is sleep-driven with SPT and the time up to assessment influencing the speed of attention, and to some extent the ‘very good’ regularity index. In more detail, for the sleep parameters with highest contribution, findings were suggestive for longer SPT, especially for older children in this subsample, to decelerate the speed of attention (also visualized in Fig. 2). In addition, for older children in this subsample specifically, assessment far from BT decelerated their speed of attention, and assessment far from their SM improved their consistency in attention. Note for example, that an individual can have a slow reaction time (slow speed) and this for several consecutive sets (high consistency).Whereas for younger children in this subsample, speed of attention decelerated when assessment was closer to WU, SM and BT (scatterplots not shown). In terms of the ‘very good’ regularity of attention index, especially in the older children shorter SPT, and in the younger children also the assessment closer to WU, SM and BT increased the index. Interpretatively, apart from SPT, within this age-range, timing during the day of a task is relevant.

In pre-adolescents (age 9.4 ± 0.8 years [min. 8 to max. 10.9]; 50.5% girls) sleep and attentional performance as assessed with the Stabilo were unassociated.

For adolescents (age 13.4 ± 1.2 years [min. 11 to max. 18]; 51.3% girls) similar results were obtained with the standard version of the Stabilo [31.3% shared variance between sleep and attention; Chi²(54) = 128.5, p < 0.0001; lambda prime: 59.9%] and the long version of the Stabilo [31.1% shared variance between sleep and attention; Chi²(54) = 144.8, p < 0.0001; lambda prime: 56.2%]. Both associations were mostly sleep-driven. The most important contributors to their shared variances were SPT, SM, sleep irregularity (or social jetlag) as well as norm index and ‘very good’ precision index. Although the contribution to the shared variance of these parameters is nearly equivalent, their sign is opposite, and therefore suggestive that the duration of the task is another important factor in performance. In more detail (scatterplots not printed) for the sleep parameters with strongest contribution to the shared variance, the following was found: Long SPT and large positive sleep irregularities (i.e., more sleep on weekends) at any age, and especially around the age of 13–14 (i.e. crossing point Fig. 2), were associated with a higher norm index. The norm index also increased with later SM for the younger ones and earlier SM for the older ones in this subsample. Alternatively, a long SPT at any age on the standard version, and especially for the older ones on the longer version, were associated with a lower ‘very good’ precision index. On the 2-minutes version, especially earlier SM for the older ones in the subsample decreased the ‘very good’ precision index. On the 10-minutes version, earlier SM at any age was associated with a lower ‘very good’ precision index. Increased sleep irregularity at any age, and especially shorter sleep duration during weekends in the younger ones, were associated with a lower ‘very good’ precision index. Interpretatively, within this age-range, precision of attentional performance is especially relevant. The correlograms (Fig. 3a–c) may assist in the interpretation if the final root is understood as their aggregated result. An interpretative summary (Fig. 2, orange and yellow dots) would be that a non-optimized sleep to the individual need for the older ones in the subsamples adversely affects their performance on a 2-minutes attention-demanding task, whereas in younger ones it would be their 10-minutes performance.

### A posteriori-stratification

The results in the adolescent age-range were suggestive for the influence of chronotype and therefore we included the pre-adolescents into the adolescent sample (i.e., ≥8 year olds, Fig. 2 grey dots). The assessment with the standard version of the Stabilo in this sample suggested a differential associative profile (although with caution, this could also be inferred from glancing at the correlograms).

Both sleep and attention variables almost equally contributed to the shared variance of 45.0% [Chi²(54) = 289.54, p < 0.00001; lambda prima: 50.1%, Table 2]. Principal determinants of this association were SPT, SM, sleep irregularity and time since BT as well as all attention variables. The norm index and the ‘very good’ precision index showed the highest factor loadings.

In more detail (Fig. 2 shows a trend by the negative canonical scores between the variables sets) for the sleep parameters with strongest contribution to the shared variance, long SPT associated with poorer precision, regularity, speed and consistency, especially in the older children (scatterplots not printed). An earlier SM also adversely affected attention performance especially in the older children. Yet for younger children (in the ≥8 years old subsample) specifically the regularity of attention was poorer with later SM. With irregular sleep, attentional performance especially deteriorated in the youngest children such that more sleep during the weekend was associated with a poorer norm index, and less sleep on the weekend worsened precision and regularity of attentional performance (scatterplots not printed). Sleep irregularity (or here social jetlag) such as sleeping more during week or more during weekends was related to slower reaction times in young children (i.e., higher best speed index). Whereas from age 10 and older, this irregularity may improve the consistency in their attentional performance. Assessment far from BT was associated with better precision and regularity especially in the older children. Whereas their speed and consistency of attention were better with assessment close to BT. Note that around the age of 10–11 years old canonical scores cross the zero-line in Fig. 2.

An interpretative summary here would be that across the age-ranges in this subsample (≥8 years old) particularly SPT, SM, sleep regularity and BT have their adverse impact on a 2-minutes attention-demanding task. The correlograms (Fig. 3d) and the gradual declining canonical scores as shown in Fig. 2 similarly depict this.

## Discussion

The findings from this study provide evidence that regular sleep is imperative for fast, precise and steady attentional performance. These behavioral states share 40–45% of the variance with performance being sleep-driven. That is, not only sleep duration but also sleep regularity and sleep midpoint were significant determinants, especially from pre-adolescence onwards. In younger children sleep was important for the speed of attentional performance and, possibly, with the manifestation of chronotypes, individual differences in attentional performance emerged. As a result, the regularity of sleep, the individual sleep midpoint and bedtime become increasingly important, across developmental stages, for optimal and steady performance throughout the day. These findings have important implications for the acquisition of skill and performance in childhood, as well as for an approach to the sleep habits of youngsters.

The first main finding is that during childhood the substantial shared variance of sleep with attentional performance is sleep-driven. This underscores the importance of defining and measuring optimal sleep during childhood. That is, the field of pediatric sleep medicine should be more critical regarding the variety of methods that exist for measuring sleep^34,50^. Consensus guidelines and panel groups as well as critical self-reflection on applied sleep (research) methodology is indeed compulsory at the verge of a sleep health era. Despite the complexity of operationalizing the sleep behavior of an individual in their habitat, it is furthermore a societal responsibility to provide opportunities for sleep throughout childhood. Contrariwise, we should particularly remain cognizant that in the absence of objective sleep assessment, and in the realm of the dispute about adequate pediatric sleep duration^4^, that our sample might be (chronically) sleep deprived.

The second main finding relates to the importance of the developmental stage concerning childhood sleep, and its impact on performance. On the one hand, the proportion of unexplained variance in this sleep-attentional performance relationship almost doubles from young childhood to adolescence. In school-aged children, complex and interactive relationships among school schedules, parental sleep-wake patterns, socioeconomic status, and daytime activities exist in determining the schedules of children^51^. Such societal characteristics, potentially interacting with intrapersonal factors, adversely affect bedtime, sleep onset time and total sleep time, especially in adolescence^52^. On the other hand, our findings on the role of sleep irregularity (social jetlag) and the time elapsed since sleep midpoint suggest the emergence of diverse somnotypologies from puberty onwards. There is furthermore reasonable indication that part of the unexplained variance could be ascribed to gender-specific alterations in sleep-wake states since canonical correlation models did not converge in girls and boys separately. For example in the literature, girls were found to be more morning-oriented than boys^53^. In other words, which sleep parameter phenotypes your sleep the best? Together, our findings may stimulate a ‘pediatric’ interpretation of the two-process model first proposed by Borbély^54,55^ which has been primarily useful in describing normal sleep in adults^56^. Namely, the prominence of the two main processes affecting sleepiness, i.e. circadian rhythms and homeostatic sleep drive, may shift around pre-adolescence, and hence affect their performance. Gender differences have been well acknowledged in psychology or neurosciences, yet the role of sleep might have been overlooked.

The third main finding concurs with the small to moderate correlations between sleep and performance in general as reported in the literature, and with the inconsistent results on which neurocognitive and neurobehavioral performance is (mostly) affected. Namely, impaired performance might be due to such MLA as assessed in our study. MLA as a potential ‘latent’ dysfunction might act in a cumulative or synergetic manner to the assessed performance. Although in the current study we did not investigate separate neuropsychological functions, our attention-demanding task involved a letter encoding, visual search and motor transform (and auditory feedback) activation of the brain. Their combined output, however, reflected the allocation of attentional resources in terms of different indexes. In the younger children, primarily speed of attention and, in the older children, stability of attention were changed by sleep. In the aforementioned experimental designs^12,14–16,19–22^ deteriorating reaction times of the neuropsychological functions investigated, as well as behaviors suggestive of inattention and sleepiness, were reported, as in a true cause-effect relation. Our findings therefore add to the existing literature that, given the concept of a default-mode network brain activity, these experiment-derived results might be moderated and/or mediated by mind-wandering. This might be (mistakenly) perceived as fatigue, sleepiness or sleep inertia by scientists, clinicians, or significant others, or in neurosciences as inefficient or slower information processing^17^. Our findings may therefore further support the notion by Carskadon *et al*.^12^ that children, during the formative period of their brain networks, may not recover from sleep restriction as rapidly as adults.

The fourth main finding is that only one root consistently reflected the shared variance, and this consequently suggests the importance of considering the activation states of the cortex in their entirety. Future research may move beyond conceptualizations in terms of time-on-task or time-of-day as regards performance variables, or sleep timing, duration, and midpoint, as regards sleep variables. The association between sleep ‘parameters’ and performance might not be simplistic. Presumably, critical points could be around the age-ranges of 8–10 years and 13–14 years. Although additional studies are needed, if one root reflects the activation states of the cortex, then studies should investigate the brain’s default-mode network activity across childhood and sleep researchers may consider studying sleep longitudinally.

The present study has several limitations. Habitual sleep was not objectively recorded; moreover, SPT, as calculated in our study rather reflects the time allocated to sleep than actual sleep. Regarding the subjective report of sleep patterns, the discrepancy between parent versus adolescent reported sleep patterns is undocumented. We also lack other (self-)measures of circadian preference^25^ which might be different from the applied chronotype formula. Next, we have no measure of sleepiness, chronicity of poor sleep, or sleep disorders in this sample. Future studies should therefore focus on objective measurement of inter- and intra-individual differences in sleep and acrophase, in addition to self-/parental reported sleep measures. Somnotypology may lead to better understanding of the interdependence between the sleep-states and waking-states default brain activity, especially regarding performance during the next day. Another limitation might be that the Stabilo assessment was performed in small groups of 3–5 children, simultaneously during schooldays, at the time most convenient for their school schedule. Yet, Wilson *et al*.^57^ showed that a 5-minute psychomotor vigilance task in a primary school classroom setting was reliable. Apart from gender, we did not pursue subgroup analysis based on grades in school or socio-economic status. Lastly, given the lack of objective sleep recordings we did not discuss the neural substrates^35,58,59^ potentially underlying our findings.

The present study has also several strengths including the age-range, the inclusion of the time elapsed since known sleep determinants, and especially an objective measure sensitive to MLA. We also did not conceptualize circadian preference in a bi-dimensional construct of owls and larks, but applied an approximation of chronotype based on parental reported hours. Furthermore, the separate sleep and attentional parameters were analyzed together, capturing their interdependence. Lastly, in contrast to others we assessed a large pediatric sample in their naturalistic environment.

In summary, the findings of this study replicate and extend an established literature on the importance of acquiring adequate sleep and of maintaining a sleep schedule that is consistent with the individual biological sleep need across childhood. Alternatively, while an increasing number of studies have actively manipulated sleep in order to observe neurocognitive and behavioral consequences, they may have consistently overlooked the role of momentary lapses of attention. Therefore sleep and waking, and their characteristic states, may benefit from a more holistic research approach.
